# Alternative oxidase promotes high iron tolerance in *Candida albicans*

**DOI:** 10.1128/spectrum.02157-23

**Published:** 2023-11-06

**Authors:** Rishabh Sharma, Andrew A. Gibb, Kelcie Barnts, John W. Elrod, Sumant Puri

**Affiliations:** 1 Oral Microbiome Research Laboratory, Kornberg School of Dentistry, Temple University, Philadelphia, Pennsylvania, USA; 2 Department of Cardiovascular Sciences, Cardiovascular Research Center, Lewis Katz School of Medicine at Temple University, Philadelphia, Pennsylvania, USA; 3 Oral and Maxillofacial Pathology, Medicine and Surgery, Kornberg School of Dentistry, Temple University, Philadelphia, Pennsylvania, USA; Stony Brook University, Stony Brook, New York, USA

**Keywords:** *Candida albicans*, iron, mitochondria, reactive oxygen species, alternative oxidase

## Abstract

Although crucial for cellular functioning, iron is toxic when present in excess. Iron overload can create a labile iron pool (LIP) that harms the mitochondrion, resulting in disruption of oxidative respiration. Fungal pathogen *C. albicans* possesses an unconventional terminal oxidase [alternative oxidase (AOX)] that can disrupt the electron transport chain to remove excess electrons, generated from various stressors, to shield the respiratory apparatus. Here, we identify a novel role for AOX under high iron, whereby it works as “safety valve” to protect the mitochondrion from high iron-induced reactive oxygen species (ROS). *C. albicans* showed pronounced metabolic flexibility to environmental iron, ranging from 1 to 500 µM. High iron significantly increased intracellular iron, LIP, and levels of cellular ROS. Expression of both *AOX1/2* was induced by iron, and cells lacking Aox1/2 showed high levels of mitochondrial ROS as well as significant growth defect under high iron. Thus, Aox1/2 helps mitigate the negative effects of iron. Furthermore, iron-induced expression of *AOX1/2* was intermittent, thus preventing ATP loss despite AOX being non-proton motive. Mitochondrial oxygen consumption rate (OCR) was significantly higher (62.8%) under high iron conditions. Contribution of Aox1/2 was greater toward this high OCR, compared to their contribution in low iron conditions. Lastly, enhanced virulence of *C. albicans* that is observed for wild-type cells under high iron was lost when fungal cells lacking *AOX1/2* were used for murine infection. Thus, AOX is crucial for preserving mitochondrial function for survival in a high iron host.

**IMPORTANCE:**

The yeast *C. albicans* exhibits metabolic flexibility for adaptability to host niches with varying availability of nutrients including essential metals like iron. For example, blood is iron deplete, while the oral cavity and the intestinal lumen are considered iron replete. We show here that *C. albicans* can tolerate very high levels of environmental iron, despite an increase in high iron-induced reactive oxygen species (ROS) that it mitigates with the help of a unique oxidase, known as alternative oxidase (AOX). High iron induces *AOX1/2* that limits mitochondrial accumulation of ROS. Genetic elimination of *AOX1/2* resulted in diminished virulence during oropharyngeal candidiasis in high iron mice. Since human mitochondria lack AOX protein, it represents a unique target for treatment of fungal infections.

## INTRODUCTION

C*andida albicans* is the most common human commensal fungus. It causes opportunistic mucosal and systemic infections in immunocompromised individuals (1). Virulence determinants of *C. albicans* are modified by various host factors including availability of nutrients such as iron (2). Within the human host, *C. albicans* encounters varying levels of iron in different niches. Higher levels of easily available iron are present in the oral cavity (3) and the intestinal lumen, while the levels on skin and in blood are extremely low (4 – 6). As a result, *C. albicans* has developed metabolic plasticity in terms of its ability to survive under both low and high iron conditions.

Iron plays a pivotal role in fungal pathogenesis (7). High iron positively affects various virulence factors from adherence to biofilm formation and hyphal induction in *Candida* spp. (8 – 10). The ability to grow at high iron potentially allows *C. albicans* to efficiently exploit its high iron-induced virulence mechanisms. Thus, it is not surprising that a higher incidence of fungal infections has been recorded in high iron individuals with iron overload from hemochromatosis or from chemotherapy (11), as opposed to low iron anemics.

Synthesis of several important cellular components such as DNA, lipids, sterols, as well as iron-sulfur (Fe-S) cluster and heme proteins require iron. However, survival at high iron levels comes with a cost for most living organisms, since iron is also notoriously toxic when present in excess. Iron-induced toxicity is due to its ability to participate in Fenton and Harber-Weiss reactions (12) to generate reactive oxygen species (ROS) that can damage key biomolecules and ultimately cause cell death (13). Mitochondrion is at the center of these conflicting roles of iron in biology, since it represents a site of both high iron demand as well as enhanced potential for iron-induced toxicity. Iron is a critical component of several electron transport chain (ETC) enzymes involved in oxidative respiration and is also involved in heme biosynthesis as well as biogenesis of Fe-S cluster proteins within the mitochondria (14). On the other hand, mitochondrion is also a key source of cellular ROS, since various stress conditions cause excessive mitochondrial ROS production due to electron leakage from its respiratory chain complexes (15, 16).

For regulation of cellular ROS levels, *C. albicans* cells use various detoxification mechanisms, consisting of superoxidase dismutases (SODs), catalase (CAT1), and a glutathione/thioredoxin system (17). In addition, *C. albicans* also possess two isoforms of an alternative oxidase (AOX) (Aox1 and Aox2), a secondary terminal oxidase found in the mitochondrial respiratory chain of plants, (18, 19), as well as some fungi (20, 21) and protozoa (22, 23). Dissimilar to the ETC protein complexes related to classical oxidative phosphorylation (OXPHOS), AOX is non-proton motive and hence does not produce ATP (24). By re-routing electrons away from the ETC, it helps prevent over-reduction of the downstream electron carriers that would result in generation of high levels of ROS in the vicinity of the mitochondria (25).

In various fungi, *AOX* expression is augmented under a variety of stress conditions such as osmotic stress (26), heat shock stress (26, 27), and oxidative stress (26, 28 – 32). AOX has also been shown to be important for virulence of certain pathogenic fungi (33, 34). In *C. albicans*, *AOX1/2* occur in a tandem arrangement, with *AOX2* located 1.3 kb upstream of *AOX1*, in the same transcriptional direction (32). *AOX2* contains a continuous open reading frame (ORF) of 1,098 bp, encoding a polypeptide of 365 amino acids with a calculated molecular mass of 41,940 Da. On the other hand, *AOX1*’s ORF comprises of 1,140 bp and translates into a polypeptide of 379 amino acids, with a calculated molecular mass of 43,975 Da. The predicted amino acid sequence of both genes exhibits 65.2% identity. *AOX1* is expressed constitutively, while *AOX2* is inducible in response to oxidative stress inducers like H_2_O_2_ or uncouplers of conventional ETC, such as cyanide (32), suggesting differential regulatory mechanisms. However, location of these genes in tandem, with the inducible *AOX2* upstream of the constitutive *AOX1*, suggests that the genes may also have overlapping roles. Thus, AOX plays a significant role under different stress conditions. High iron is a major producer of oxidative stress. Whether AOX provides protection against high iron-induced oxidative stress to promote *C. albicans* survival in a high iron host or iron-rich niches within a host is not known.

In the present study, we underscore the ability of *C. albicans* to survive under very high levels of environmental iron. To understand mechanisms that support this adaptability, oxidative stress mitigation systems of *C. albicans* were evaluated as a function of iron. Besides the usual protective candidates, namely, SODs and CAT, we identified a novel role for AOX as an on/off switch required to protect the mitochondria from high iron-induced ROS. We further show that activation of non-proton motive AOX under high iron is transient in nature, thereby preventing loss of mitochondrial ATP output. Furthermore, a double knockout mutant of *AOX1* and *AOX2* showed significantly reduced tongue fungal burden during oropharyngeal candidiasis (OPC), compared to its parent strain, in high iron mice. Thus, we present here a novel role for AOX in promoting fungal survival in a high iron host.

## RESULTS

### *C. albicans* shows robust growth and ATP levels under high iron, regardless of elevated ROS

**Fig 1:**
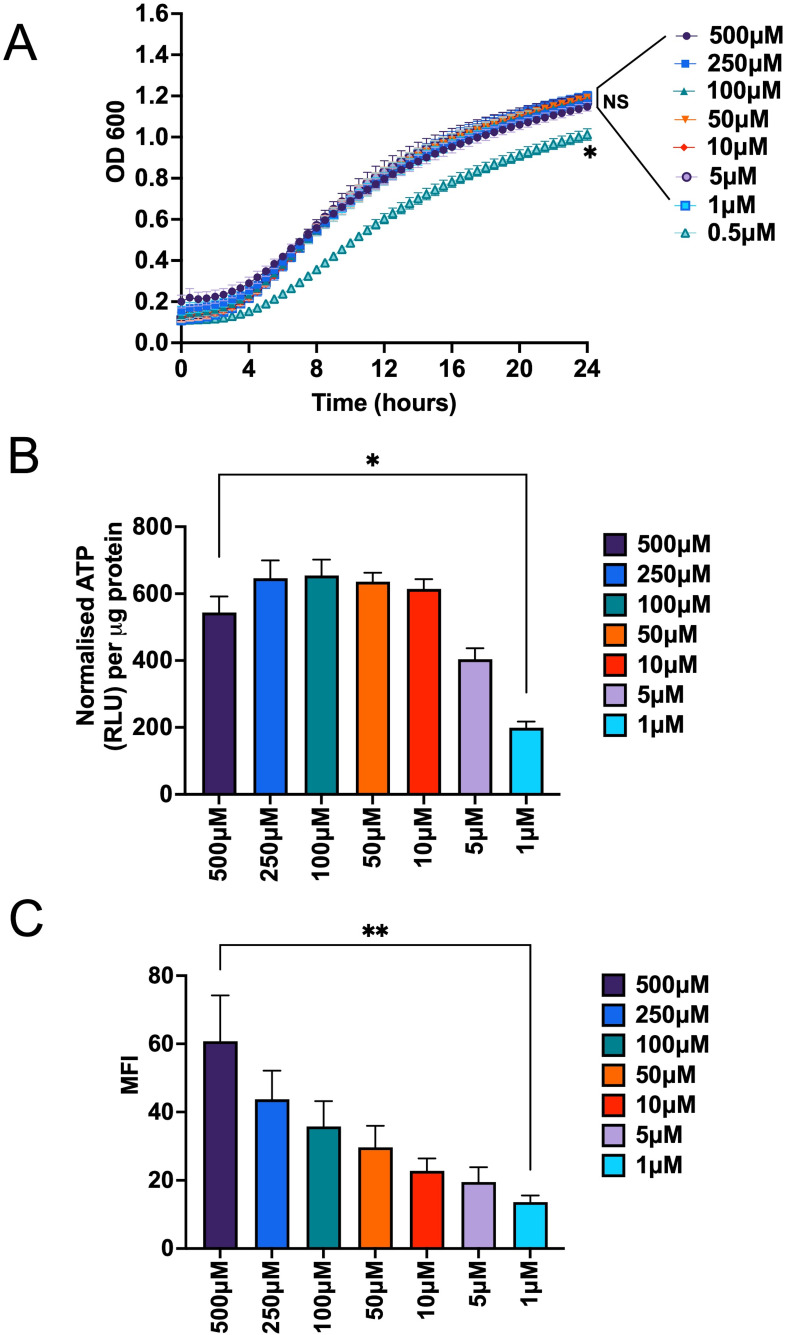
*C. albicans* growth and energy production is unaffected by high iron-mediated cellular ROS. (**A**) *C. albicans* cells was exposed to various iron concentrations (0.5–500 µM), and growth was monitored at OD_600_ over a period of 24 hours. Results of three biological repeats with four replicates each are represented as mean ± standard errors of the means (SEM). (**B**) ATP levels were measured as relative light units (RLU) in different iron concentrations (1–500 µM) by the luminescent detection assay kit and normalized to total protein concentration (micrograms). Results of four biological repeats with triplicates are represented as mean ± SEM. (**C**) ROS production was measured by a fluorescence microscopy with H2DCFDA staining. Mean fluorescence intensities (MFI) for *n* > 150 cells from two independent biological experiments are represented as mean ± SEM. Statistical significance analysis was assessed by Mann-Whitney test (*P  <  0.05; **P < 0.01; NS, statistically not significant).

To evaluate iron adaptability of *C. albicans*, growth was assessed in a yeast nitrogen base (YNB) minimal medium with varying iron concentrations (0.5 µM to 500 µM). Spectroscopic measurement showed that intracellular iron in *C. albicans* cells increased in a dose-dependent manner, with significantly higher levels (1,238-fold) observed in cells grown at 500 µM of iron, as compared to 0.5 µM iron cells (Fig. S1A). Fungal growth at all iron concentrations was ideal, except at 0.5 µM, with concentrations from 1 µM to 500 µM showing similar and overlapping growth patterns (Fig. 1A). Thus, for this study (unless otherwise stated), 1 µM and 500 µM were chosen as the respective low- and high iron conditions that did not affect growth rates. Adequate growth must be supported by adequate levels of cellular energy, and hence, we next assessed intracellular ATP levels under different iron conditions. ATP levels increased steadily from 1 µM to 10 µM of iron, reaching a saturation at 10 µM. From 10–500 µM iron, ATP levels averaged at 610.8 relative light units per micrograms of protein. Although there was a slight drop in ATP levels between 250 µM and 500 µM iron, ATP levels at 500 µM iron were significantly higher (~2.52-fold) in comparison to levels at 1 µM iron (Fig. 1B).

We next assessed labile iron pool (LIP) levels that represent the non-protein bound iron serving as a catalyst in iron-mediated Fenton reaction (35). LIP levels steadily increased in a dose-dependent manner, albeit only up to 50 µM iron, while showing saturation in levels from 50 to 500 µM iron (Fig. S1B). ROS levels, however, continued to increase in response to increasing iron concentrations. Significantly higher (4.45-fold) ROS levels were observed at 500 µM, when compared to cells grown at 1 µM iron (Fig. 1C). These results show that even at very high iron levels that generate excessive amounts of cellular ROS, *C. albicans* growth and metabolism remain largely unaffected.

### High iron induces the expression of *C. albicans AOX* genes

**Fig 2:**
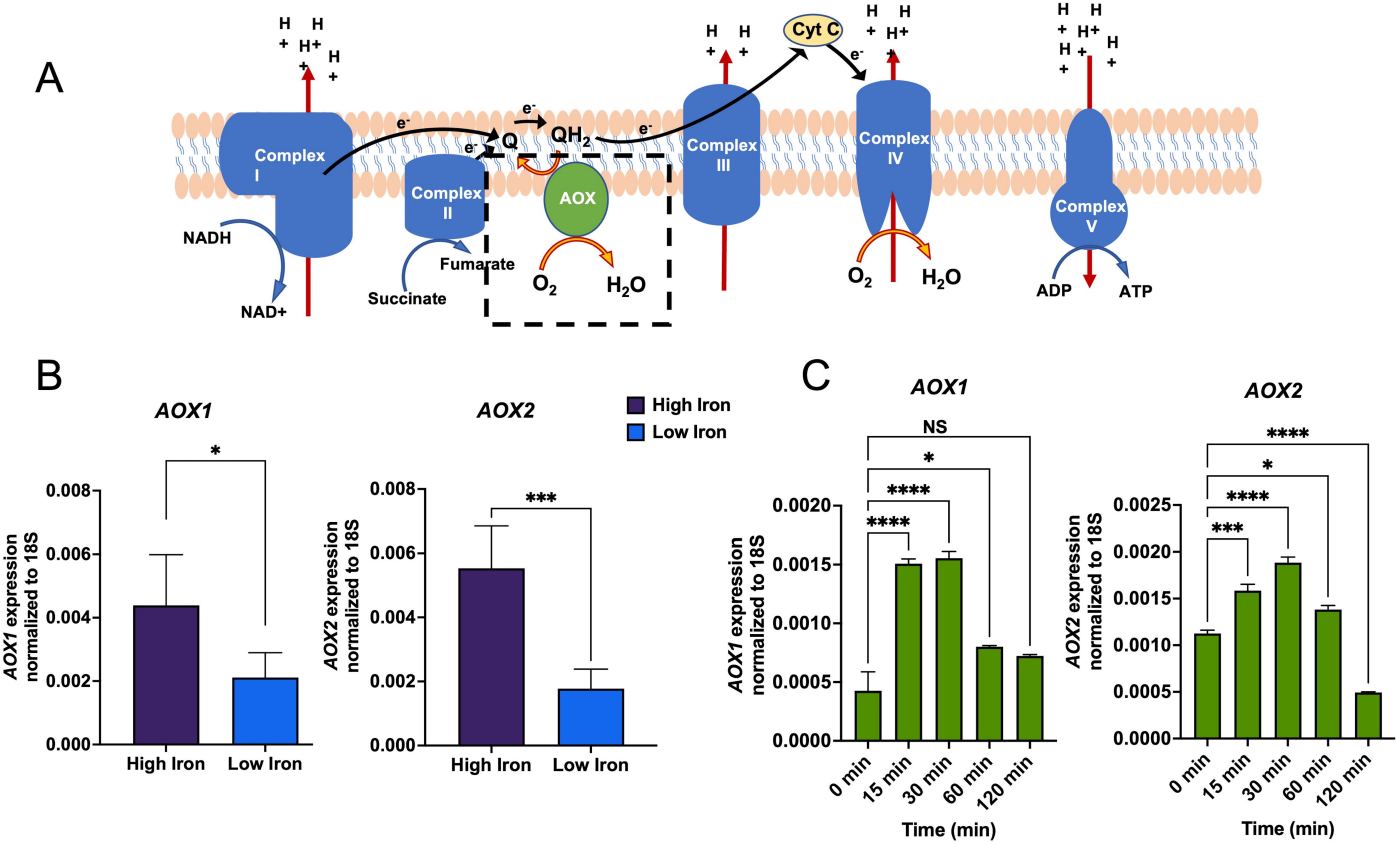
High iron induces intermittent expression of *C. albicans AOX1/2*. (**A**) Schematic representation of the fungal mitochondrial electron transport system. (**B**) *AOX1* and *AOX2* expressions were assessed at high and low iron (500 µM and 1 µM, respectively) by quantitative real-time PCR analysis. The results of four independent biological repeats with triplicates are represented as means ± SEM. (**C**) Time course of gene expression was also measured in high iron (500 µM) over a period of 120 min. Results represent mean ± SEM, from three replicates. Statistical significance analysis was assessed by paired *t* test and one-way analysis of variance (*P  <  0.05; ***P < 0.001; ****P < 0.0001; NS, statistically not significant).

To evaluate the role of the conventional antioxidant systems in protecting the fungal cells from ROS, we performed a gene expression analysis of *C. albicans CAT* and *SOD*s under low and high iron. As expected, most of the genes that are conventionally involved in oxidative stress mitigation, namely, *CAT1*, *SOD1*, *SOD2*, *SOD3*, and *SOD6*, showed upregulation (4.7-, 5-, 5.3-, 2-, and 2.4-fold, respectively) under high iron (Fig. S2). Since AOX locally protects the mitochondria from ROS by providing an alternative route for electrons (31, 36) (Fig. 2A), we hypothesized that *C. albicans* Aox1/2 can potentially play an important role in protecting the mitochondria from high iron-mediated ROS. Both *AOX1* and *AOX2* were significantly upregulated (2.1- and 3.13-fold, respectively) under high iron (Fig. 2B). We further observed that high iron-induced temporal expression of *AOX1* and *AOX2* was transient in nature (Fig. 2C).

### AOX alleviates mitochondrial ROS

To specifically test the ability of Aox1/2 in mitigating iron-induced mitochondrial ROS accumulation, we grew the following strains under low and high iron (to stain them with MitoSOX, a mitochondria-specific superoxide indicator): *aox1*/*aox1 aox2*/*aox2* mutant strain and its respective parent strain (CAI4 + *URA*), along with *aox1*/*aox1 aox2*/*aox2* strain reconstituted with *AOX2* (*aox1*/*aox1 aox2*/*aox2 + AOX2*).

**Fig 3:**
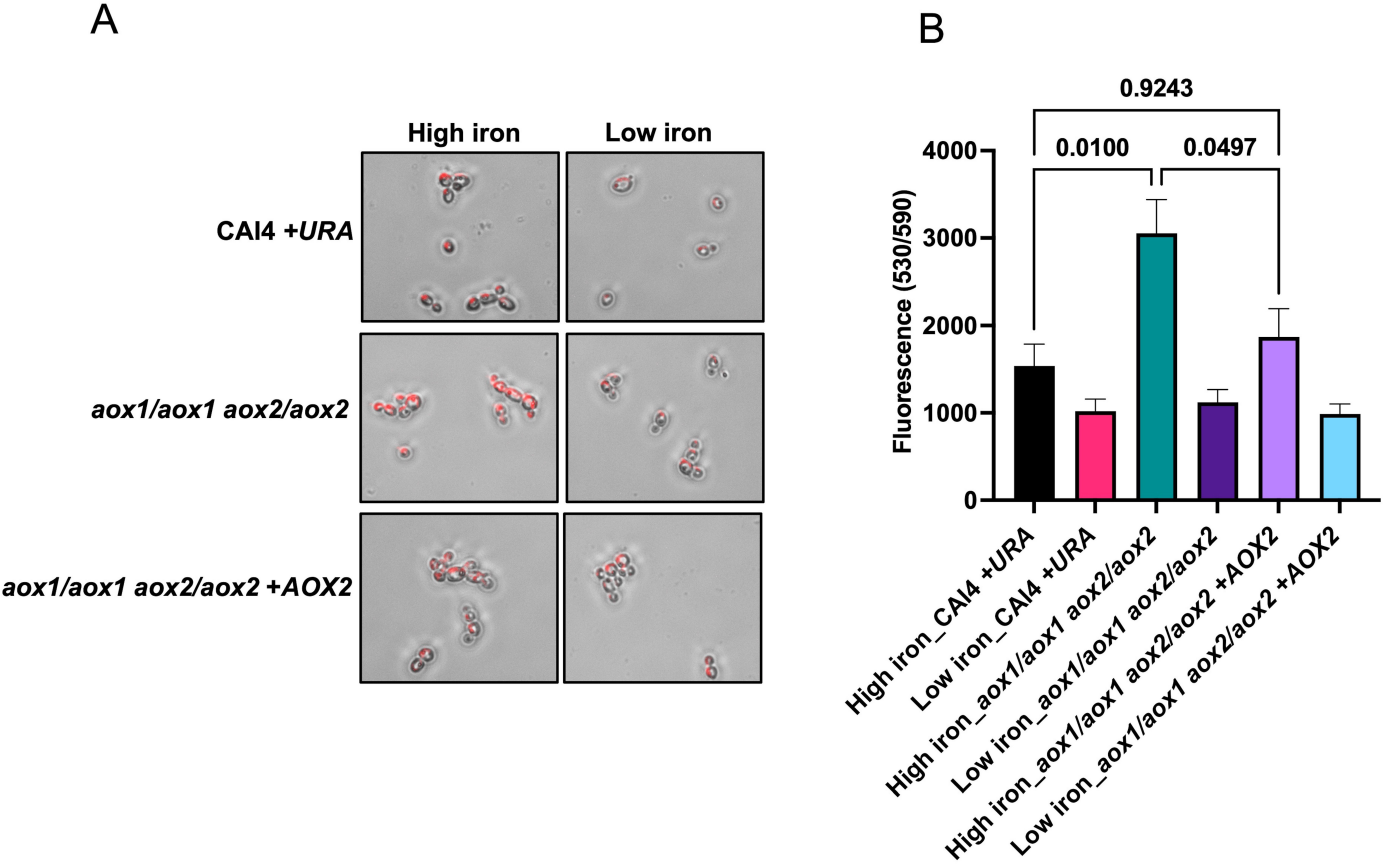
Mitochondrial ROS was increased in *aox1*/*aox1 aox2*/*aox2* cells grown under high iron conditions. (**A**) Log phase *C. albicans* cells (grown in high and low iron; 500 µM and 1 µM, respectively) were incubated with 5 µM MitoSOX Red as a marker for mitochondrial superoxide and imaged by fluorescence microscopy. (**B**) MitoSOX Red fluorescence was measured spectrophotometrically at excitation/emission of 530 nm/590 nm with an 80% gain by BioTek Synergy multi-mode reader where fluorescence intensity was measured as the signal obtained with 1 × 10^7^ cells. The results of four independent biological repeats with triplicates are represented as means ± SEM. Significance analysis was assessed by one-way analysis of variance.

Cells grown under high iron showed significantly higher mitochondrial ROS, when compared to cells grown under low iron, for all three strains. However, across all strains and growth conditions, mitochondrial ROS levels were the highest in *aox1*/*aox1 aox2*/*aox2* cells grown in high iron (Fig. 3A and B). Reintegration of *AOX2* in this mutant strain restored ROS levels to those observed for its parent strain, under high iron. Therefore, AOX is crucial in preventing mitochondrial ROS accumulation in *C. albicans* cells growing under high iron conditions.

### AOX supports *C. albicans* growth under high iron

**Fig 4:**
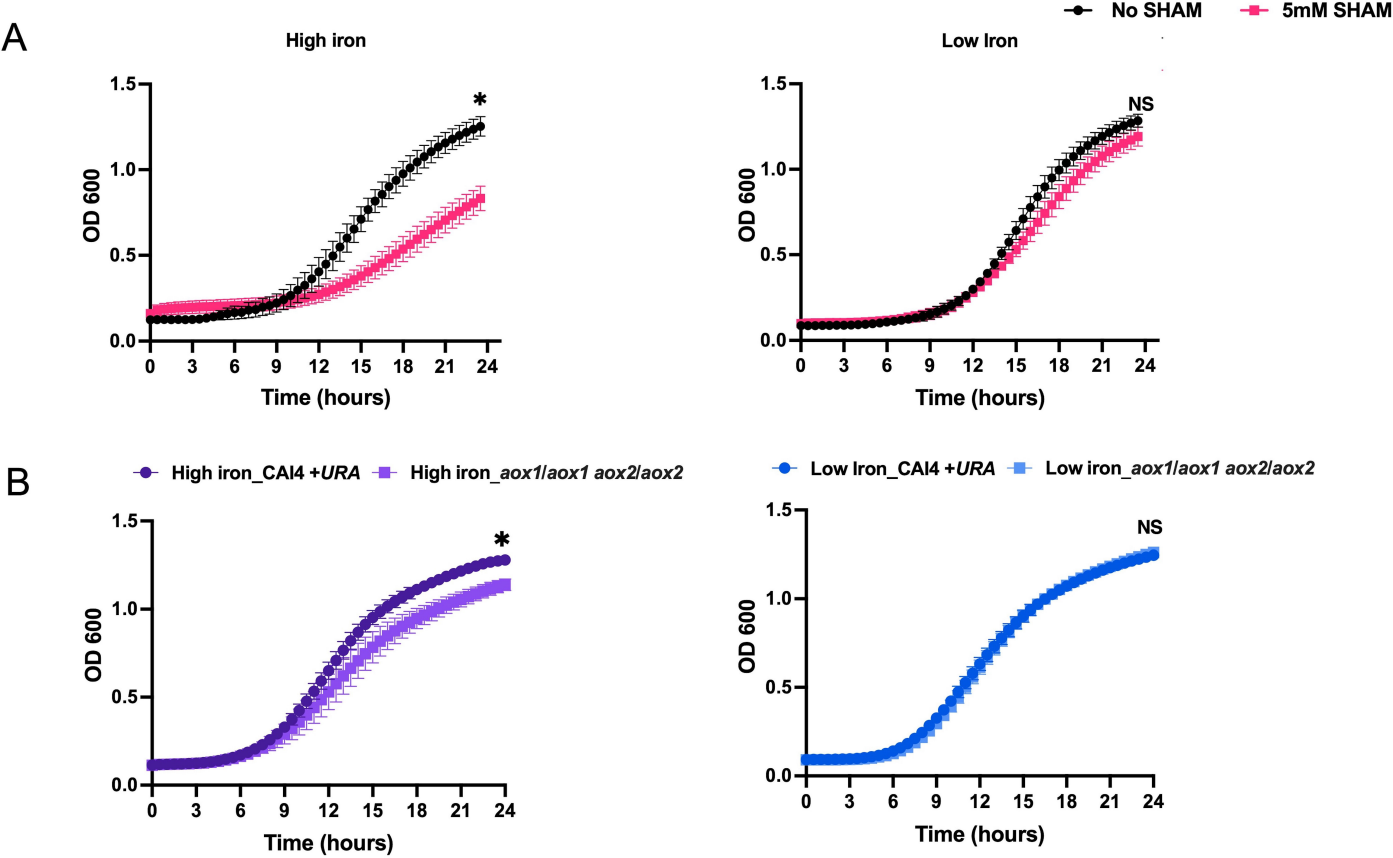
Aox1/2 are crucial in maintaining *C. albicans* growth under high iron conditions. (**A**) Growth curve of WT *C. albicans* under high (500 µM) and low iron (1 µM) with or without 5 mM SHAM inhibitor. (**B**) Growth was also assessed under high and low iron for *aox1*/*aox1 aox2*/*aox2* cells, along with its parent strain (CAI4 + *URA*). The results of three independent biological repeats with triplicates are represented as means ± SEM. Significance analysis was assessed by Mann-Whitney test (*P  <  0.05 ; NS, statistically not significant).

To further test the importance of Aox1/2 for growth under high iron, we compared the growth rates of (i) untreated wild-type (WT) *C. albicans* with WT cells treated with AOX inhibitor [salicylhydroxamic acid (SHAM)] (Fig. 4A) and (ii) *aox1*/*aox1 aox2*/*aox2* mutant with its respective parent strain (Fig. 4B) under low and high iron. WT cells grown in the presence of SHAM (compared to WT cells without SHAM) as well as *aox1*/*aox1 aox2*/*aox2* mutant (compared to its parent strain) showed significantly lower growth rates in high iron [*P* value = 0.001 (Fig. 4A) and 0.028 (Fig. 4B), respectively]. Under low iron, similar growth rates were observed between both pairs of strains, suggesting that AOX plays an important role in supporting *C. albicans* growth only under high iron.

### AOX provides a greater contribution toward the elevated mitochondrial respiration under high iron

**Fig 5:**
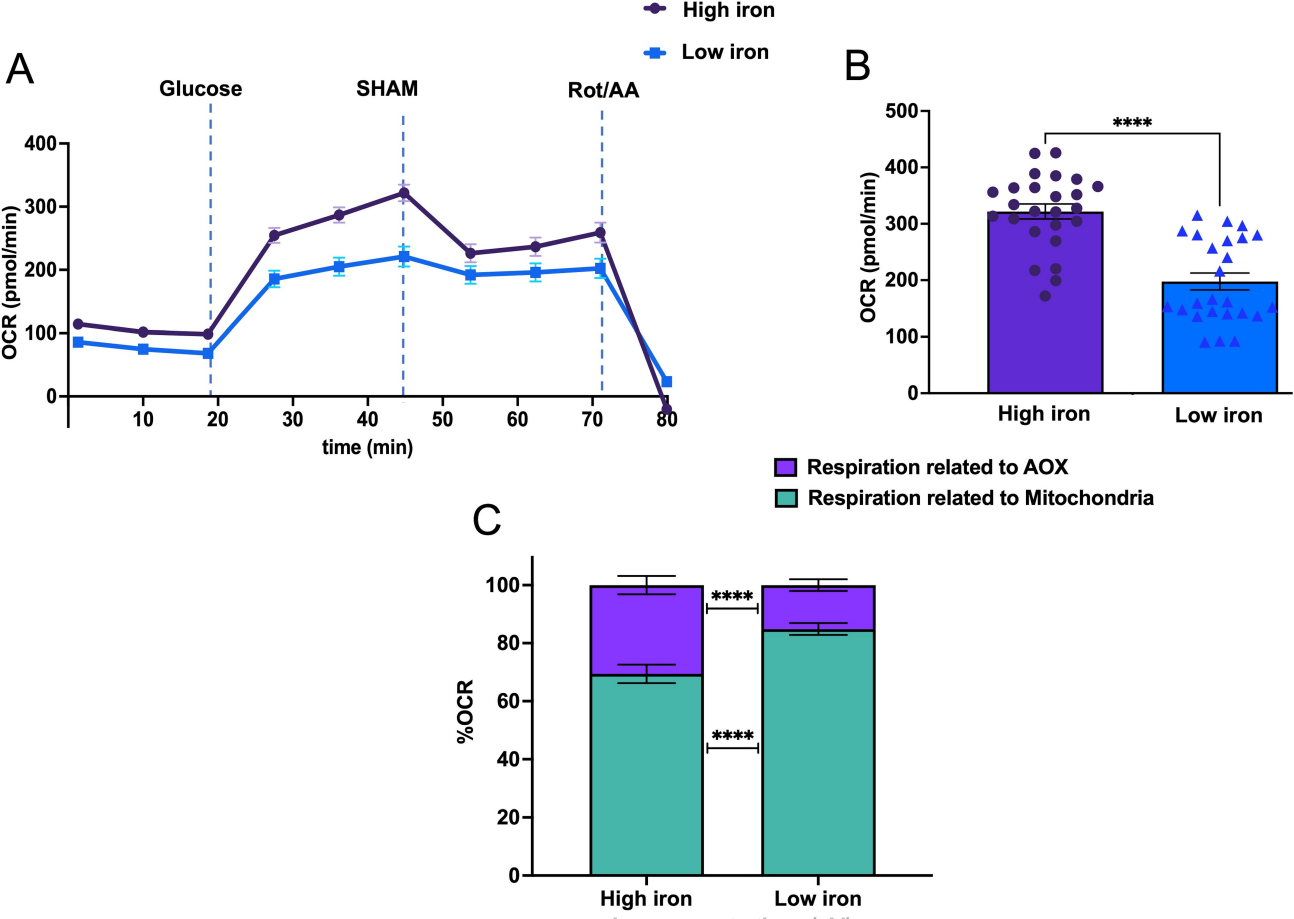
High iron causes increased mitochondrial OCR that has a greater contribution from AOX. (**A**) Schematic representation of Seahorse analysis to measure OCR. *C. albicans* OCR was measured over a course of 80 min using XF96 extracellular flux Seahorse analyzer. Glucose (10 mM), SHAM (5 mM), and rotenone (Rot, 1.5 µM) along with antimycin A (AA, 10 µM) were sequentially delivered to wells containing *C. albicans* in high (100 µM) or low (1 µM) iron YNB medium (pH 7.4) through injection ports in sensor cartridge. Dark violet and blue lines represent the average value of multiple time point measurements for high iron and low iron, respectively. (**B**) Quantification of OCR value from the Seahorse analyzer for total mitochondrial contribution attained at the peak of respiration after glucose addition, right before addition of SHAM. (**C**) Percent OCR values related to total mitochondrial respiration as well as total respiration without the contribution of AOX (measured after addition of SHAM). Data were pooled from three independent experiments, and bars show the mean ± SEM. Significance analysis was assessed by Mann-Whitney test; ****P  <  0.0001.

We next assessed *C. albicans* oxygen consumption rate (OCR), an estimation of mitochondrial respiration in real time, under low and high iron, using a Seahorse Extracellular Flux analyzer (Fig. 5A). OCR was significantly increased (62.8%) under high iron conditions, compared to low iron group in the presence of 10 mM glucose (Fig. 5B). To identify percent OCR specifically attributable to Aox1/2, as opposed to the conventional mitochondrial OXPHOS machinery, OCR was also measured in the presence of the AOX inhibitor SHAM (Fig. 5A). Under high iron conditions, 30.5% of the total mitochondrial oxygen consumption was attributable to Aox1/2, whereas only 15.1% was contributed by Aox1/2 under low iron (Fig. 5C). In support of efficient ATP generation under high iron (Fig. 1B), these data suggest that the mitochondria function at a higher efficiency in high iron cells, compared to cells grown in low iron conditions, and AOX activity has a greater contribution to overall mitochondrial respiration under high iron.

### AOX is crucial for high iron-mediated increase in virulence during OPC

**Fig 6:**
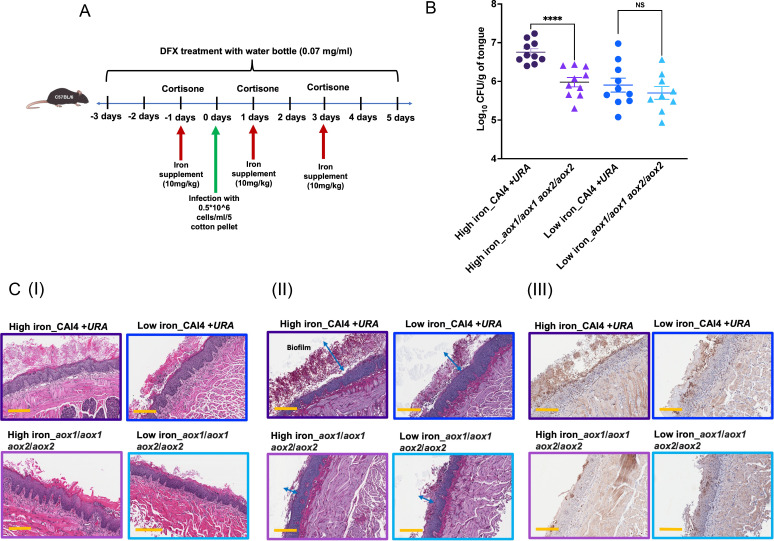
*aox1*/*aox1 aox2*/*aox2* cells showed reduced virulence in high iron mice with OPC. (**A**) Pictorial representation of high and low iron murine OPC model. In the high iron mouse group, iron dextran was administered intraperitoneally at a dose of 10 mg/kg on days −1, 1, and 3. Conversely, the low iron mouse group was treated with deferasirox at a concentration of 0.07 mg/mL in drinking water containing 2% dextrose from days −3 to 5. (**B**) CFU/gram of tongue tissue was obtained from *C. albicans* infected mice. The data were pooled from two independent experiments and presented as mean ± SEM. Statistical analysis was performed using Mann-Whitney test (****P ≤ 0.0001 ; NS, statistically not significant). (**C**) Tongue tissue inflammation, fungal load, and neutrophil activity were assessed by staining with hematoxylin and eosin (**I**), Periodic-acidic Schiff (II), and myeloperoxidase (III), respectively. Tissue sections (4 µm thick) are displayed at 10× magnification of mice tongue. Blue arrows indicate *C. albicans* biofilm thickness, and yellow scale bars shows a size of 200 µm.

To determine the role of AOX in *C. albicans* virulence in a high iron host, we infected mice with parent strain and *aox1*/*aox1 aox2*/*aox2* cells, using our low and high iron murine model of OPC (Fig. 6A) (37). High iron mice infected with *aox1*/*aox1 aox2*/*aox2* cells showed a significantly reduced tongue fungal burden, compared to those infected with the parent strain (*P* = 0.0001; Fig. 6B). However, this strain-specific difference (between *aox1*/*aox1 aox2*/*aox2* strain and its parent strain) in fungal burden was lost in low iron mice that showed similar tongue fungal burdens for both strains.

Furthermore, sagittal tongue tissue sections of infected mice were stained with hematoxylin and eosin (H&E), Periodic acid-Schiff (PAS), and myeloperoxidase (MPO) stains [Fig. 6C; (I), (II), and (III), respectively]. Tongues of high iron mice infected with parent strain cells showed a markedly thickened stratum corneum that was composed of organized and stratified sheets of keratin [Fig. 6C (I)] and a larger fungal biofilm mass on the tongue surface, with numerous candida hyphae and spores inhabiting the prominent keratin layer [Fig. 6C (II)]. Neutrophil infiltration of the tongue epithelia that occurs because of infection was also most prominent in high iron mice infected with the parent strain cells [Fig. 6C (III)]. However, all of these features of enhanced virulence in high iron mice with parent strain infection were not observed in high iron mice infected with *aox1*/*aox1 aox2*/*aox2* cells, with the latter resembling parent strain infection of low iron mice (Fig. 6C). Thus, AOX contribution is crucial in maintaining the enhanced virulence of *C. albicans* that is observed in a high iron host.

## DISCUSSION

Iron levels in the human host can vary greatly, from extremely low levels approaching 10^−12^ µM in blood (38) to 400 µM in the gastrointestinal tract (GI) (39). Our iron concentrations of up to 500 µM (Fig. 1A) provide direct evidence for the ability of *C. albicans* cells to withstand such high levels of environmental iron. However, free iron levels in saliva can easily exceed its total iron binding captivity (40), and iron levels in the intestinal lumen can potentially also reach toxic levels due to enhanced bioavailability at acidic pH of the intestinal lumen (6). Our results show that *C. albicans*, which is a common commensal in the oral cavity and the lower GI, is well equipped to handle this toxicity, as shown by the concomitant increase in expression of *CAT* and *SOD* genes (Fig. S2).

While iron-dependent induction of *CAT1* has been previously shown (41), it was also observed that *SOD4* is induced upon iron starvation (42). Induction of *SOD1* and *SOD5* has been correlated to oxidative stress, albeit independent of iron (43, 44). However, this is the first study to reveal the extensive network of high iron-mediated induction of multiple *SOD* genes (Fig. S2) in *C. albicans*. Among these protective enzymes, only Sod2 is located within the mitochondria (45, 46), the organelle most susceptible to ROS (47). Surprisingly, however, *C. albicans* was observed to have enhanced mitochondrial activity (ATP yield and OCR; Fig. 1B and 5B) in high iron, despite higher iron-mediated ROS (Fig. 1C), underscoring the existence of additional unknown mechanisms that shield the mitochondria during high iron growth.

We discover here that *C. albicans* AOX mediates a novel mechanism required for preserving mitochondrial function under high iron, since cells lacking *AOX1/2* had excessive mitochondrial ROS accumulation, leading to growth defects under high iron (Fig. 3 and 4). Previous studies in fungal systems including *C. albicans* have highlighted the role of AOX in managing mitochondrial ROS, and growth defects under oxidative stress were observed in its absence (31, 46, 48). Here, we showed how high environmental iron specifically induces *AOX1*/*2* expression and that AOX is important for *in vitro* and *in vivo* growth under high iron (Fig. 4A, B, and 6B), thus establishing a unique role for AOX in *C. albicans*.

A challenge of AOX-mediated control of ROS is that it comes with a cost of ATP loss, owing to the non-proton motive nature of AOX (24, 49). Thus, our observation of no significant loss in ATP (Fig. 1B) at high iron that led to *AOX1*/*2* induction (Fig. 2B) was intriguing and potentially is a result of the fact that iron induction of *AOX1*/*2* in *C. albicans* was intermittent in nature (Fig. 2C). We propose that *C. albicans* AOX functions as an on/off “safety valve” that is activated upon encountering high iron-mediated ROS and is shut off until ROS levels increase again in response to high iron. Only other known example of intermittent expression of AOX was previously reported in the ascomycetes fungus *Magnaporthe grisea*, where the gene expression was noted to be labile, rapid, and transient (30). AOX expression is regulated by various mechanisms in stresses, including transcriptional (30) and post-transcriptional mechanisms (50, 51), such as protein degradation and modification. Mechanisms responsible for this unique expression style in *C. albicans* will require further investigation.

Previous studies suggest a close relationship between mitochondrial respiration and *C. albicans* infection and virulence (52). Similarly, we observed growth defects for cells lacking Aox1/2 in high iron mice during OPC (Fig. 6B), and these defects *in vivo* were more drastic than those observed *in vitro* (Fig. 4A and B). The host innate immune response exhibits fungicidal activity by enhancing macrophage- and neutrophil-mediated ROS production (oxidative burst) to trigger inflammasome activation (1, 53). Cumulative effects of high iron-mediated ROS and host innate immunity-mediated ROS seem to exacerbate oxidative insults *in vivo*, thus making AOX crucial for virulence in a high iron host.

Fungal infections are hard to treat, and the incidence of drug resistance is increasing (54). A study found that 13% of the elderly (ages 67 to 96) had high body iron stores (55), and 1 in 10 Caucasians are heterozygous for a common mutation that causes the genetic iron overload disease (hemochromatosis) (56). High iron is also considered to be a major etiology for diabetes (57). Since most of the above-mentioned groups are also highly susceptible to candidiasis, targeting AOX opens newer avenues in the treatment of fungal infections in this set of individuals.

## MATERIALS AND METHODS

### Fungal strains, media, culture conditions, and animals

*C. albicans* strains used in this study are as follows: SC5314 (WT, prototrophic clinical isolate) (58) and Ura+ *aox1*/*aox1 aox2*/*aox2* double mutant strain (∆*ura3::imm434*/∆*ura3::imm434* ∆(*aox1b-aox1a*)::*hisG*/∆(*aox1b-aox1a*)::*hisG-URA3-hisG*) (32, 59), along with its parent strain CAI4 + *URA* (*ura3Δ::imm434/URA3*) (60). In addition, an *AOX2* restoration strain (*ura3::imm434*/∆*ura3::imm434* ∆(*aox1b-aox1a*)::*hisG*/∆(*aox1b-aox1a*)::*RPS10 Δrps10::CIp10-AOX2-URA3*) was created (as described in the following section), whereby an *AOX2* allele was reintegrated into a Ura− version of *aox1*/*aox1 aox2*/*aox2* double mutant strain, restoring the Ura status after integration.

A 1.7 g/L of minimal YNB media without copper, ammonium salt, and iron (4027-112; MP Biomedicals) supplemented with 2.5 µM copper sulphate (CuSO4), 5 g/L ammonium sulphate (NH_4_SO_4_), 2% glucose, 0.79 g/L amino acid supplement (Complete Supplement Mix: 4500-012, MP Biomedicals), and 50 µM of iron chelator bathophenanthriline-disulfonic acid (146617; Sigma) was used as basal medium (61). Various concentrations of ferric chloride (FeCl_3_·6H_2_O) were then added to achieve respective iron concentrations. For all experiments, exponential-phase *C. albicans* cells were obtained from two subsequent overnight cultures in the respective iron medium, as described in Tripathi et al. (10). All experiments were performed at 30°C.

Experiment involving animals utilized 4- to 6-week-old C57BL/6 female mice sourced from Jackson Labs, Bar Harbor, ME. Animals were treated humanely in accordance with the protocols approved by Temple University (IACUC project no. 5079).

### Generation of *AOX2* reintegration strain

To create a Ura− version of *aox1/aox1 aox2/aox2* strain, 1 × 10^7^ of Ura+ *aox1/aox1 aox2/aox2* cells were plated on YNB agar plate with 1 mg/mL 5-fluoroorotic acid (Sigma) and 50 µg/mL uridine, eliminating *URA3* gene-containing colonies (62). We confirmed uracil auxotrophy on uridine-deficient YNB agar plates.

A PCR-amplified *AOX2* allele was then introduced into the aox1*/aox1 aox2/aox2* (Ura− version) using the CIp10 vector, a *URA3* integration plasmid that allows chromosomal integration of the gene of interest into the *RPS10* locus for expression under *RPS10* promoter (63). Briefly, full-length *AOX2* gene was PCR amplified, digested with XhoI/EcoRV enzymes, and ligated into the CIp10 vector. After linearization with NcoI, the linear fragment was introduced into the *aox1/aox1 aox2/aox2* strain (Ura− version) using the Frozen-EZ Yeast Transformation II Kit (Zymo Research, CA, USA). Transformed cells were plated onto YNB agar plates lacking uracil, enabling the selection of *URA*+ colonies of Ura*+ aox1/aox1 aox2/aox2 + AOX2* strain. To confirm the integration of *AOX2*, genomic DNA from reintegration strain was extracted and confirmed by PCR amplification using primers specific to *AOX2*.

### Assessment of growth

To assess growth under different iron conditions, *C. albicans* cells were cultured in respective iron media for two subsequent overnight cultures, as described above. Cells from the second overnight culture were diluted to 0.1 optical density at 600 nm (OD_600_) in 200 µL of fresh respective media in a 96-well polystyrene plate (655 180, Greiner Bio-One). The plate was further incubated at 30°C, and growth curve was assessed by measuring absorbance at 600 nm over 24 hours, every 30 minutes, using a BioTek Synergy Multi Mode Reader.

To understand the role of AOX on growth, WT *C. albicans* growth was assessed in the presence or absence of 5 mM SHAM (S607, Sigma), an AOX inhibitor, or growth of *aox1/aox1 aox2/aox2* mutant and its parent strain, CAI4 + *URA*, was assessed, under high and low iron conditions, in a 96-well polystyrene plate, as discussed above.

### Measurement of ATP levels

Intracellular ATP levels under different iron conditions were determined as described previously in Tripathi et al (10). Briefly, *C. albicans* cells grown in different iron conditions were harvested at 5,000 rpm, washed twice with PBS, and resuspended in 500 µL of PBS containing protease inhibitor cocktails (11836153001, Sigma). Cell lysates were prepared by bead beating with glass beads, using Fast Prep-24 (MP Biomedicals). The cell debris was removed by centrifugation at 13,000 rpm for 5 minutes. The amount of protein in cell lysates was measured using the bicinchoninic acid (BCA) assay (23227, Thermo Scientific). After normalizing protein levels, cell lysates were used for ATP quantification using ATP assay kit (A22066, Invitrogen) according to the manufacturer’s instructions. One hundred microliters of the reaction solution (containing luciferin-luciferase) was added to 100 µL of cell lysate in 96-well black microtiter plate (3916, Corning). The results were presented as relative light units representing average ATP levels normalized to total protein concentration.

### Intracellular ROS assessment

Intracellular ROS levels were assessed using 2′,7′-dichlorodihydrofluorescein diacetate (H2DCFDA) (C2938, Invitrogen) staining and confocal microscopy, as described previously (10). *C. albicans* cells from two subsequent overnight cultures in different iron conditions were diluted to 0.3 OD_600_ in fresh respective medium and cultured for 4 hours at 30°C. The cells in the logarithmic growth phase were subsequently collected by centrifugation and resuspended in PBS. After adjusting for equal cell numbers in PBS, 10 µM of H2DCFDA was added, and cells were incubated at 30°C with shaking at 180 rpm for 30 minutes. Following incubation, cells were pelleted, washed twice with PBS at 8,000 rpm, and re-diluted in 100 µL of PBS for imaging. Fluorescence images were taken with a confocal microscope using an argon laser (510 or 520 nm lines) in combination with FITC filters. The images were quantified using ImageJ software. The results were presented as mean fluorescence intensity for *n* > 150 cells from two independent biological experiments.

### Inductively coupled plasma optical emission spectroscopy (ICP-OES)

Total intracellular iron in *C. albicans* cells grown in varying iron concentrations was evaluated, as previously described (37). Briefly, *C. albicans* cells were cultured in various iron conditions for two consecutive overnight periods in metal-free tubes (3194-335-001-9, Labcon). On experimental day, cells were diluted to 0.3 OD_600_ in their respective iron medium for 4 hours, and logarithmic growth phase culture was pelleted, washed thrice, and normalized to OD_600_ to obtained equal number of cells that were stored in −80°C. Frozen samples were digested using Nitric acid (Ultrex purity; Fisher Scientific, Waltham, MA) and transferred to a graphite heating block (Environmental Express, Charleston, SC). Next, samples were heated and digested at 95°C for 30 minutes. To prepare the samples for trace metal analysis, samples were spiked with an internal standard solution containing scandium, indium, and praseodymium to a final concentration of 5 ng/mL and diluted to 5 mL with deionized water. Iron concentration in exposed samples was determined using ICP-OES with a Thermo Fisher iCAP 7600. The iron emission signal in the sample extracts was monitored in axial mode at λ = 239.56 nm.

### Measurement of LIP

The amount of LIP in *C. albicans* cells grown under different iron conditions was determined by treating cells with Calcein AM (C1430; Invitrogen) (64). Calcein AM-loaded cells exhibit a fluorescence that is quenched by intracellular iron. Briefly, *C. albicans* cells grown for two subsequent overnight cultures in different iron conditions were diluted to 0.3 OD_600_ in fresh respective iron medium and cultured for 4 hours to obtain cells in logarithmic growth phase. Cells were then centrifuged at 8,000 rpm, washed twice, and resuspended in PBS to adjust to equal cell numbers. Five micromolars of Calcein AM were added to the 1 × 10^7^ cells and incubated for 60 minutes at 30°C with gentle shaking (150 rpm) in the dark. After incubation, cells were washed three times and resuspended in PBS. Equal number of cells (0.4 × 10^7^ cells) were used to measure fluorescence intensity using a fluorescence spectrophotometer (BioTek Synergy HTX) at an excitation wavelength of 485 ± 20 nm and an emission wavelength of 528 ± 20 nm.

### Real-time quantitative PCR

Total RNA was extracted from *C. albicans* cells grown under high and low iron. RNA was isolated using Qiagen RNA isolation kit (74134, Qiagen) by bead beating in RLT lysis buffer. Briefly, 350 µL of RLT lysis buffer was resuspended with 10-mL culture pellet of high and low iron *Candida* cells grown to log phase. Next, tubes were subjected to bead beating (6–7 cycles, 6 m/s) with 0.45-mm diameter glass beads using a FastPrep-24 instrument (MP Biomedicals). Lysed cells were centrifuged to remove cell debris, and supernatant was passed through a gDNA Eliminator spin column in combination with a high-salt buffer for efficient removal of genomic DNA. Furthermore, 350 µL of 70% ethanol was added, and total RNA was purified according to the manufacturer’s instructions. Isolated RNA was confirmed for absence of DNA by a PCR amplification step using the extracted RNA as template and quantitative PCR (qPCR) primers, with genomic DNA as positive control and no template as a negative control. One microgram of DNA-free RNA sample was used for complementary DNA (cDNA) synthesis using iScript cDNA synthesis kit (1708891, Bio-Rad). Furthermore, equal volume (1 µL) of cDNA was used for determining transcript levels via reverse transcription real-time qPCR (RT-qPCR) using gene-specific primers and SYBR Green PCR Supermix (1725124, Bio-Rad). Relative quantities of the mRNAs for the genes of interest and 18s rRNA gene (housekeeping gene) were calculated from their corresponding standard curves using QuantStudio 3 real-time PCR system. Expression of gene of interest was assessed after normalization with the level of 18S rRNA gene in the same sample for each respective condition. The results were expressed as mean of triplicate samples ±  SEM.

### Assessment of mitochondrial ROS level

Mitochondrial ROS were measured using MitoSOX Red (M36008, Invitrogen), which accumulates in the mitochondrial matrix and is oxidized to a fluorescent product by superoxide. Briefly, *C. albicans* cells were grown overnight in different iron conditions for two subsequent cycles. Afterward, the cells were diluted to 0.3 OD_600_ in fresh respective medium corresponding to each iron condition and cultured for 4 hours at 30°C. The cells in the logarithmic growth phase were then collected through centrifugation and resuspended in PBS. Subsequently, cell numbers were adjusted to 1 × 10^7^ cells in respective medium containing of 5 µM MitoSOX Red dye and incubated at 30°C for 30 min. The stained cells were washed twice and resuspended in PBS for microscopic visualization of MitoSOX Red fluorescence at 40× using EVOS M5000 microscope. Furthermore, equal number of cells (0.4 × 10^7^ cells) in PBS were added to a black 96-well plate to measure fluorescence intensity using a fluorescence spectrophotometer. Fluorescence intensity was measured by BioTek Synergy multi-mode reader with excitation/emission at 530 nm/590 nm with a gain of 80%.

### Measurement of mitochondrial OCR

Mitochondrial respiration rate was measured in real time as OCR, using a Seahorse XF analyzer (XF 96), as previously described (65). Briefly, exponentially grown *C. albicans* cells in high and low iron YNB medium (pH 7.4) were washed twice with PBS. Cells were further resuspended in respective medium without glucose and adjusted to 125 K cell/well at a final volume 180 µL and added to XF 96-well microplates (102601-100, Seahorse Bioscience), pre-coated with 0.01% of Poly-L-lysine (P4832; Sigma) for 1 hour at room temperature. All cell preparations in this assay medium (without glucose) were maintained in XF 96-well microplate for 1 hour at 30°C to permit cell adhesion before analysis. Afterward, the cell plate was centrifuged at 1,500 rpm, and fresh respective medium was added to each condition. OCR was measured after addition of 10 mM glucose into port A to measure mitochondrial respiration in the presence of substrate, followed by addition of 5 mM SHAM into port B to measure the contribution of AOX to mitochondrial oxygen consumption. Finally, rotenone (R8875; Sigma)/antimycin A (A8674, Sigma) combination was injected into port C at a final concentration 1.5 µM/10 µM, respectively, to completely inhibit all mitochondrial respiration. Data were analyzed using the Wave software 2.6.3.

### Murine OPC study

Previously described immunosuppressed model of murine OPC was used (66). Briefly, C57BL/6 mice (female, 4 to 6 weeks old) were immunosuppressed by subcutaneous injection of 225 mg/kg of cortisone acetate (C3130; Sigma) on days −1, 1, and 3. In high iron mice group, mice were supplemented with iron dextran (D8517; Sigma) intraperitoneally (10 mg/kg) on days −1, 1, and 3, while treatment with deferasirox (QA-8243; CombiBlock) (0.07 mg/mL in drinking water with 2% dextrose for days −3 to 5) was used for low iron mice group.

On the day of infection (0 day), mice were anesthetized with ketamine:xylazine (10:1) and sublingually infected with 5 × 10^6^ cells/mL of either *aox1*/*aox1 aox2*/*aox2* mutant or parent strain (CAI4 + *URA*) for 45 minutes. On day 5, mice were euthanized, and tongue tissue was harvested and divided into two halves, lengthwise. One part was homogenized in PBS followed by serial dilution and plated on yeast extract peptone dextrose agar plates containing streptomycin/penicillin (SV30010; HyClone) and incubated at 30°C for 48 hours. Fungal burden in tongue tissue was presented as mean log_10_ values of CFU. Second half of tongue was fixed in 10% formalin, paraffin-embedded, and 4-µm-thick sections were prepared. Tongue tissue sections were used to assess epithelial structure, inflammatory cell infiltration, and fungal infection by staining with H&E and PAS stains previously described in Puri et al. (37). Neutrophils infiltration levels were also assessed by MPO immunostaining.

## SUPPLEMENTAL MATERIAL

Supplemental file 1 spectrum.02157-23-s0001.docx Fig. S1 and S2 and Table S1.

ASM does not own the copyrights to Supplemental Material that may be linked to, or accessed through, an article. The authors have granted ASM a non-exclusive, world-wide license to publish the Supplemental Material files. Please contact the corresponding author directly for reuse.
