# Supplementary material for: Alternative oxidase promotes high iron tolerance in Candida albicans
Source: Microbiol Spectr. 2023 Nov 6;11(6):e02157-23. doi: 10.1128/spectrum.02157-23 (PMC10714975; doi:10.1128/spectrum.02157-23)
Supplement: Supplemental file 1 — Fig. S1 and S2 and Table S1. [file spectrum.02157-23-s0001.docx]

*Supplementary Information for*

**Alternative oxidase promotes high iron tolerance in *Candida albicans***

Rishabh Sharma^a^, Andrew Gibb^b^, Kelcie Barnts^c^, John W Elrod^b^, and Sumant Puri^a*.^

^*^Corresponding author: Sumant Puri

E-mail: [sumantpuri@temple.edu](mailto:sumantpuri@temple.edu)

**Supplementary figures**


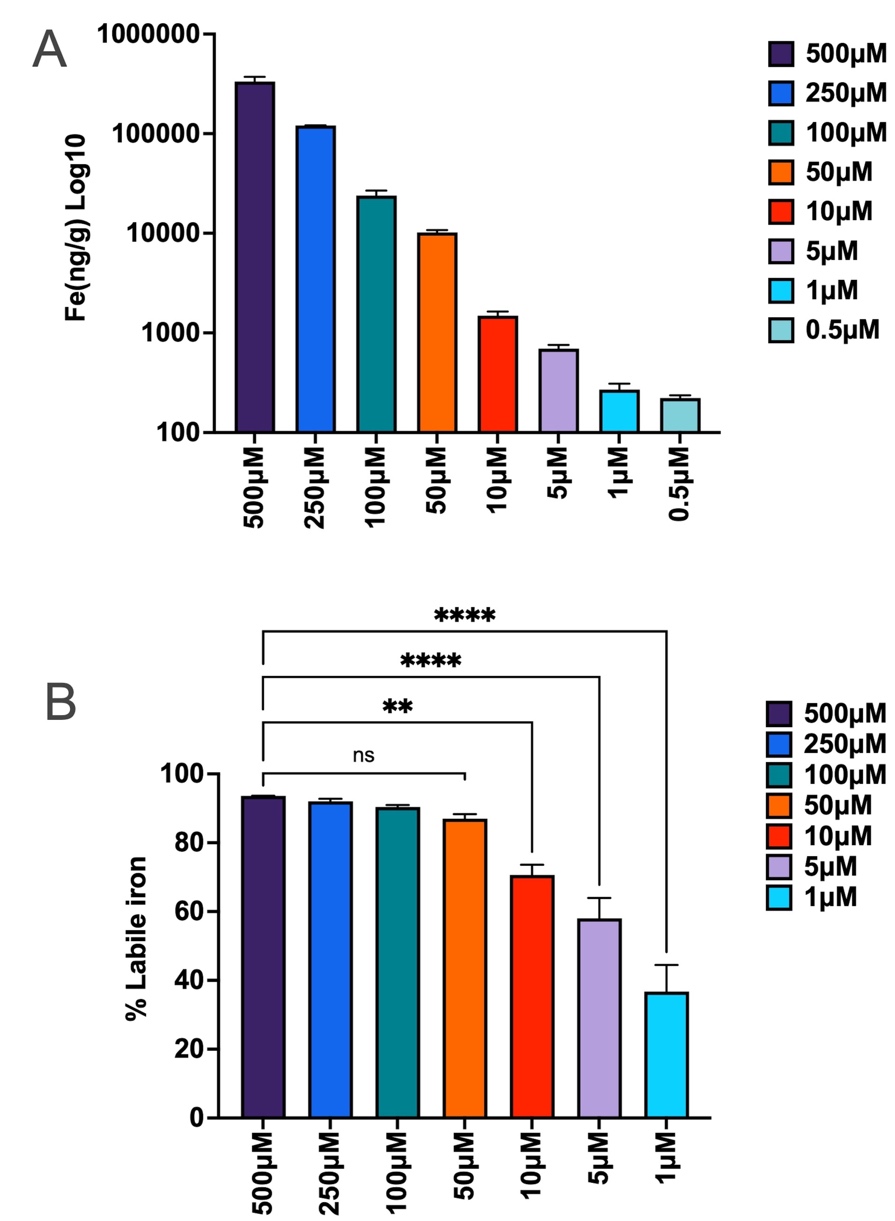


**FIG S1** Total intracellular iron level and labile iron pool (LIP). A) Intracellular iron level was measured by ICP-OES analysis. The results of triplicates are represented as means ± SEM. B) The % cellular LIP was measured using CalceinAM under different iron condition. Results represent the mean ± SEM from three independent experiments and significance analysis was done by one-way ANOVA, **<0.01, *****p* < 0.0001.


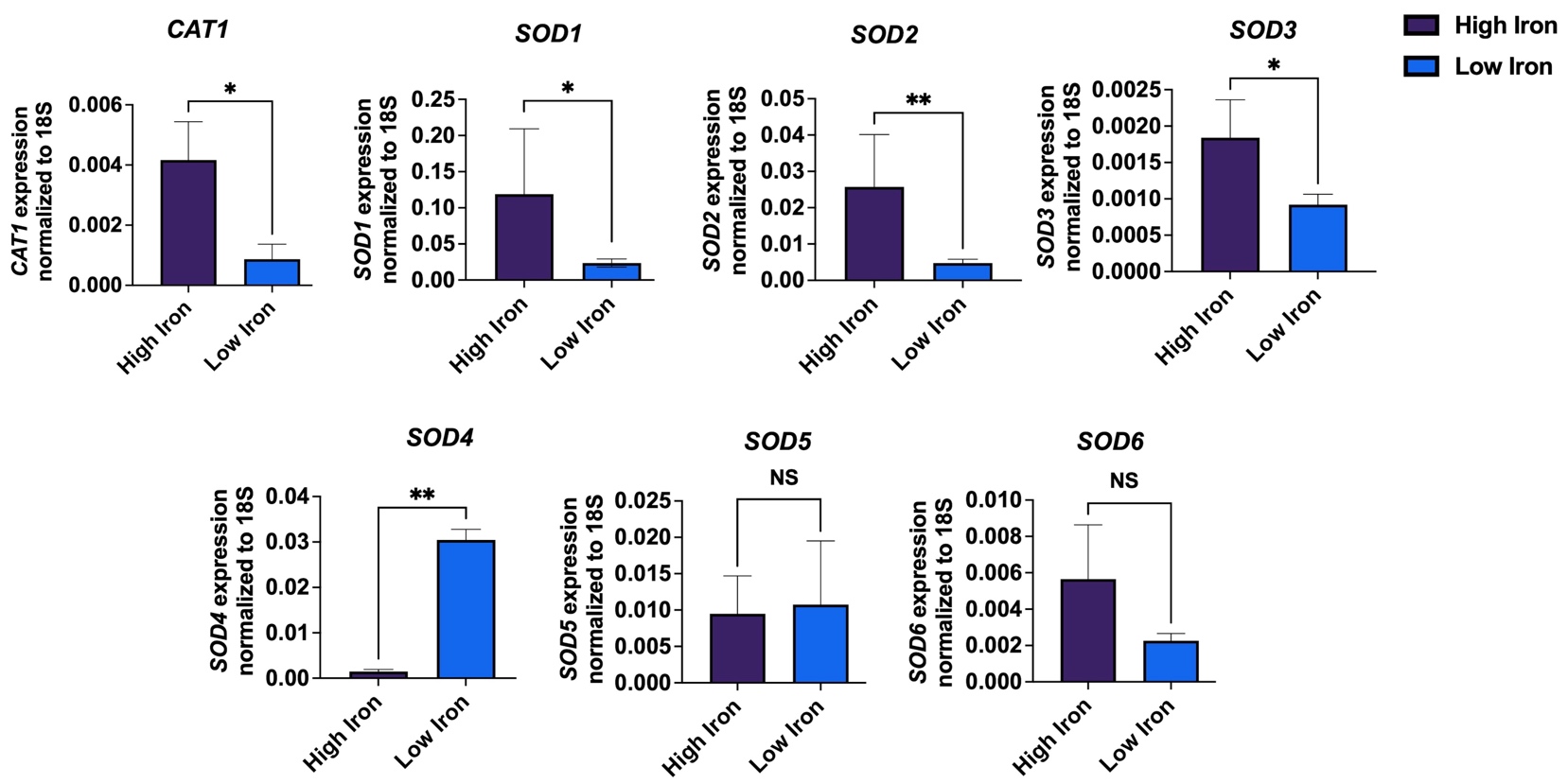


**FIG S2** Quantitative real-time PCR analysis of catalase, and superoxide dismutase genes was performed. The results of five independent biological repeats with triplicates are represented as means ± SEM*.* Statistical significance analysis was performed using Mann-Whitney U test, **p* < 0.05, **<0.01.

**Table S1 Primers used in this study.**

| **Gene** | **Primer sequence** |
| --- | --- |
| *AOX1F* | CACCACGTACCATTGGGGAT |
| *AOX1R* | AACGGAACCAGGAACACCAG |
| *AOX2F* | TTTAGCAGCACCACCACACT |
| *AOX2R* | CACTGGATGTGGATAAGGTGCT |
| *SOD1F* | TCCGAATCCGCTCCAACCACA |
| *SOD1R* | AAATGAGGACCAGCAGAAGTACAACCA |
| *SOD2F* | TCAATTGAACAAGCCGTTGAAGCCAAA |
| *SOD2R* | ACCACCTTGAGAGACAGGAGCCA |
| *SOD3F* | CAATGCCGCTATTGACGCACTTGA |
| *SOD3R* | TCCAGAACAAACTGTGGTTGGTGTGT |
| *SOD4F* | TGACTCCAAAGGCAAGGCACCA |
| *SOD4R* | TGGGCCAACACCTGAAGGCAAT |
| *SOD5F* | ACGAGGGACACGGCAATGCT |
| *SOD5R* | GCGCCATTACCTTGAGGAGCAGTA |
| *SOD6F* | GACCCCGACCCACCTCAACAA |
| *SOD6R* | GGGTAGCAAGGAGTGCCGGT |
| *CAT1F* | AGAGTTGGTCAACACGGTCC |
| *CAT1R* | CACCATAAGCACCGGAACCT |
| *18SF* | CCGGAATCGAACCCTTATTC |
| *18SR* | GCTGGCGATGGTTCATT |
| *AOX2F* Cloning | CTCGAGATGCTTACTGCTTCGCTTTACA |
| *AOX2R* Cloning | GATATCTTATAATTGTAAATCTTGTTTTTCCC |
